# β3-Adrenoceptor as a new player in the sympathetic regulation of the renal acid–base homeostasis

**DOI:** 10.3389/fphys.2024.1304375

**Published:** 2024-02-22

**Authors:** Serena Milano, Ilenia Saponara, Andrea Gerbino, Dominga Lapi, Ludovica Lela, Monica Carmosino, Massimo Dal Monte, Paola Bagnoli, Maria Svelto, Giuseppe Procino

**Affiliations:** ^1^ Department of Biosciences, Biotechnologies and Environment, University of Bari, Bari, Italy; ^2^ Department of Sciences, University of Basilicata, Potenza, Italy; ^3^ Department of Biology, University of Pisa, Pisa, Italy

**Keywords:** β3-adrenoceptor, H^+^-ATPase, PKA, acid loading, acid–base balance

## Abstract

Efferent sympathetic nerve fibers regulate several renal functions activating norepinephrine receptors on tubular epithelial cells. Of the beta-adrenoceptors (β-ARs), we previously demonstrated the renal expression of β3-AR in the thick ascending limb (TAL), the distal convoluted tubule (DCT), and the collecting duct (CD), where it participates in salt and water reabsorption. Here, for the first time, we reported β3-AR expression in the CD intercalated cells (ICCs), where it regulates acid–base homeostasis. Co-localization of β3-AR with either proton pump H^+^-ATPase or Cl^−^/HCO_3_^−^ exchanger pendrin revealed β3-AR expression in type A, type B, non-A, and non-B ICCs in the mouse kidney. We aimed to unveil the possible regulatory role of β3-AR in renal acid–base homeostasis, in particular in modulating the expression, subcellular localization, and activity of the renal H^+^-ATPase, a key player in this process. The abundance of H^+^-ATPase was significantly decreased in the kidneys of β3-AR^−/−^ compared with those of β3-AR^+/+^ mice. In particular, H^+^-ATPase reduction was observed not only in the CD but also in the TAL and DCT, which contribute to acid–base transport in the kidney. Interestingly, we found that in *in vivo*, the absence of β3-AR reduced the kidneys’ ability to excrete excess proton in the urine during an acid challenge. Using *ex vivo* stimulation of mouse kidney slices, we proved that the β3-AR activation promoted H^+^-ATPase apical expression in the epithelial cells of β3-AR-expressing nephron segments, and this was prevented by β3-AR antagonism or PKA inhibition. Moreover, we assessed the effect of β3-AR stimulation on H^+^-ATPase activity by measuring the intracellular pH recovery after an acid load in β3-AR-expressing mouse renal cells. Importantly, β3-AR agonism induced a 2.5-fold increase in H^+^-ATPase activity, and this effect was effectively prevented by β3-AR antagonism or by inhibiting either H^+^-ATPase or PKA. Of note, in urine samples from patients treated with a β3-AR agonist, we found that β3-AR stimulation increased the urinary excretion of H^+^-ATPase, likely indicating its apical accumulation in tubular cells. These findings demonstrate that β3-AR activity positively regulates the expression, plasma membrane localization, and activity of H^+^-ATPase, elucidating a novel physiological role of β3-AR in the sympathetic control of renal acid–base homeostasis.

## Introduction

The kidney tubules contribute to acid–base balance by excreting proton execess and reabsorbing filtrated bicarbonate. The bulk of the filtered bicarbonate is reabsorbed in the proximal tubule, in parallel with proton secretion in the lumen, and virtually, no bicarbonate remains in the final urine. In addition, the early portion of the distal nephron, encompassing the thick ascending limb (TAL) and the distal convoluted tubule (DCT), expresses the H^+^-ATPase and significantly contributes to acid–base transport in the kidney, even though the physiological importance of H^+^-ATPase expression in these segments remains to be clarified (Frische et al., 2018). Moreover, the phenotype of disease-causing mutations in the B1 subunit gene of the H^+^-ATPase may also relate to the presence of H^+^-ATPase in the TAL and DCT (Frische et al., 2018). The final tuning of urinary acidification occurs in the collecting duct (CD) tubule mainly through the action of the CD intercalated cells (ICCs): type A, type B, non-A, and non-B ICCs (Roy et al., 2015). A-ICCs secrete protons into urine through the apical H^+^-ATPase and H^+^-K^+^-ATPase while reabsorbing bicarbonate via the basolateral Cl^−^/HCO_3_^−^ exchanger AE1. B-ICCs show a mirrored localization of membrane transporters: they secrete bicarbonate into urine via the apical Cl^−^/HCO_3_^−^ exchanger pendrin while extruding protons through a basolateral H^+^-ATPase (Roy et al., 2015). There are also non-A and non-B ICCs with a range of functions that still remain under investigation. The kidneys are innervated by efferent sympathetic nerve fibers that directly contact the vasculature, the renal tubular epithelial cells, and the juxtaglomerular granular cells (Johns et al., 2011). Although the role of the sympathetic nervous system via beta-adrenoceptors (β-ARs) in controlling renal blood flow, glomerular filtration rate, sodium and water reabsorption, and renin secretion is widely documented (Johns et al., 2011; Procino et al., 2016; Grisk, 2017; Osborn et al., 2021), its role in modulating acid–base balance still needs to be elucidated. It has been previously established that β1-AR is expressed in intercalated cells (Boivin et al., 2001), associated with the regulation of acid–base homeostasis in distal segments of the kidney tubule (Roy et al., 2015) and that the β-AR-agonist isoproterenol can regulate ICC functions (Morel et al., 1976; Iino et al., 1981; Morel and Doucet, 1986; Hayashi et al., 1991; DiBona and Kopp, 1997). We previously reported that β3-AR is expressed in the TAL, the DCT, and the outer portion of the CD and regulates the trafficking of the water channel AQP2 (Procino et al., 2016) and the activation/function of both the sodium–potassium–chloride symporter NKCC2 (Procino et al., 2016) and the sodium–chloride cotransporter NCC (Milano et al., 2021). Here, we reported the plasma membrane expression of β3-AR in ICCs for the first time. Considering that the mammals basically cope with acidic dietary intake, this work aims to provide the first evaluation of a possible regulatory role of β3-AR in modulating the expression, localization, and activity of H^+^-ATPase in the TAL, DCT, and CD ICCs, where β3-AR is physiologically expressed. In mice, we demonstrated that β3-AR knockout significantly reduced the expression of H^+^-ATPase and blunted the kidneys’ ability to excrete proton excess in the urine during an acid challenge. Using *ex vivo* stimulation of kidney slices, we demonstrated that β3-AR stimulation promotes H^+^-ATPase apical expression in all nephron segments expressing the receptor. Moreover, we studied the effect of the β3-AR stimulation on H^+^-ATPase functional activity and the intracellular signal transduction pathway involved by measuring the intracellular pH recovery after an acid load in renal cells. Lastly, we investigated the effect of the β3-AR agonist mirabegron (MIR) on the urine excretion of H^+^-ATPase in a cohort of patients with overactive bladder (OAB) syndrome.

## Methods

### Antibodies and reagents

The β3-AR antagonist L-748,337 (cat. no. sc-204044), the human β3-AR agonist mirabegron (cat. no. sc-211912), a polyclonal antibody against β3-AR (cat. no. sc-1473), a polyclonal antibody against pendrin (cat. no. sc-50346), and a monoclonal antibody against H^+^-ATPase B1/2 (cat. no. sc-55544) were obtained from Santa Cruz Biotechnology (Dallas, TX, United States). The PKA inhibitor H89 (cat. no. B1427) was obtained from Sigma (St. Louis, MO, United States). Bafilomycin A1 (cat. no. S1413) and 8-bromo-cAMP (8Br-cAMP; cat.no. S7857) were obtained from Selleckchem (Houston, TX, United States). BCECF AM (cat. no. B1170) was obtained from Thermo Fisher Scientific (Waltham, MA, United States). The rabbit affinity-purified polyclonal antibody against human AQP2 was previously described (Procino et al., 2008). The anti-NCC antibody (cat. no. SPC-402D) was obtained from StressMarq Biosciences Inc. (Victoria, BC, Canada). Antibodies anti-NKCC2 (cat. no. AB3562P) and aldosterone (cat.no. A9477) were obtained from Merck Millipore (Billerica, MA, United States).

### Acid loading in mice

Animal studies were carried out in compliance with the recommendations in the Guide for the Care and Use of Laboratory Animals of the National Institutes of Health, the Italian guidelines for animal care (DL 26/14), and the European Communities Council Directive (2010/63/UE). The experimental procedures were approved by the Ethical Committee for Animal Experiments of the University of Pisa (permit number 656/2018-PR). Mice were maintained on a 12-h light/12-h dark cycle, with free access to water and food (Teklad 2018; Envigo, Indianapolis, IN). β3-AR^+/+^ and β3-AR^−/−^ mice littermates (Boss et al., 1999), maintained on the FVB/N genetic background, were obtained from The Jackson Laboratory (Bar Harbor, ME, United States). Experiments were conducted on male 4-month-old mice. Metabolic studies were performed in individual metabolic cages. Blood was collected from the tail vein before the metabolic cage study. After 5 days of monitoring in metabolic cages, β3-AR^+/+^ and β3-AR^−/−^ mice were divided into two groups: one group (control, CTR) remained on water (n = 6 for each genotype) and the second group was switched to a drinking solution containing 280 mM NH_4_Cl for 5 days to induce acid loading (n = 6 for each genotype), as previously described (Amlal et al., 2004). After 5 days, the mice were sacrificed, and blood samples were collected by cardiac puncture. *A priori* sample size calculation was performed using G*Power software version 3.1. Considering that the data analysis was performed comparing urine measurements from each mouse before and during water or NH_4_Cl watering, we calculated the sample size for two experimental groups (CTR and NH_4_Cl) using the “difference between two dependent means (matched pairs)” statistical test. The primary outcome used to determine the sample size was the urine pH. With a significance level set at 5%, power at 90%, and effect size at −2, the number of mice needed in this study was calculated as six for each group.

#### Urine analyses

Urine and plasma pH were measured using the Jenway 3510 pH meter with a micro P XS sensor electrode. Titratable acidity was determined according to Chan (1972). Ammonium concentration in the urine was measured using the phenol/sodium hypochloride method described by Berthelot (1859).

### Immunofluorescence

Mouse kidneys were fixed over night in 4% paraformaldehyde at 4°C, cryopreserved in 30% sucrose/PBS for 24°h, and embedded in an optimal cutting temperature medium. Thin cryosections (6 μm) were subjected to immunofluorescence analysis, as previously reported (Procino et al., 2016). The mouse kidney sections were incubated over night with the primary antibodies anti-AQP2, anti-H^+^-ATPase, and anti-pendrin. Cryosections of the fixed kidney from β3-AR^+/+^ and β3-AR^−/−^ mice were incubated over night with the primary antibodies anti-H^+^-ATPase, anti-AQP2, anti-NKCC2, and anti-NCC. The appropriate Alexa Fluor-conjugated secondary antibodies (Life Technologies) were used, according to the manufacturer’s instructions. Immunofluorescence experiments on human kidney samples were performed as previously reported (Milano et al., 2023). Images were acquired using a confocal laser scanning fluorescence microscope (Leica TSC-SP2, Mannheim, Germany). Each experiment was repeated at least three times. The fluorescence intensity of H^+^-ATPase labeling in the mouse TAL, DCT, and CD was performed on digital images acquired using the same exposure parameters. LAS X AF software (Leica) was used to select the areas corresponding to the H^+^-ATPase-associated fluorescence. The mean fluorescence intensity of every area was measured. A total of 120 confocal images (10 for each mouse) were blindly analyzed for both genotypes. Tubules with the lumen not clearly visible were excluded from the analysis. The final calculation included an average of 180 TAL per β3-AR^+/+^ and 185 TAL per β3-AR^−/−^, 143 DCT per β3-AR^+/+^ and 137 DCT per β3-AR^−/−^, and 157 CD per β3-AR^+/+^ and 160 CD per β3-AR^−/−^. Summary data are expressed for each group as means ± SEM.

### Western blotting

Whole kidneys isolated from β3-AR^+/+^ (*n* = 6), β3-AR^−/−^ (*n* = 6), β1,2-AR^+/+^ (*n* = 6), and β1,2-AR^−/−^ (*n* = 6) (Rohrer et al., 1999) mice were homogenized in the RIPA buffer, as previously described (Procino et al., 2014). 30 μg of proteins from each lysate were separated by SDS-PAGE using Mini-PROTEAN^®^ TGX Stain-Free™ Precast Gels (Bio-Rad) and analyzed by Western blotting. Densitometry was performed using Image Lab™ 6.1 software (Bio-Rad) after normalization for the total protein content using the Stain-Free™ imaging technology (Bio-Rad), according to the manufacturer’s instructions.

### Kidney slices

Six C57BL/6J male mice were used for the kidney slice experiments. The mice were anesthetized with isoflurane 1.5% (v/v) and sacrificed by cervical dislocation. Kidneys were collected, and thin transversal slices (250 μm) were obtained using a McIlwain Tissue Chopper (Ted Pella Inc.; Redding, CA, United States). The slices were left at 37°C in Dulbecco’s Modified Eagle Medium/F12 medium (CTR) or stimulated for 40 min with mirabegron (10^–8^ M) given alone or after 30 min of preincubation with either L748,337 (10^–7^ M) or H89 (10^–5^ M). As positive controls of the cell responsiveness, kidney slices were also treated with 8-bromo-cAMP (5 × 10^−4^ M) or aldosterone (2 × 10^−7^ M) in the presence of MIR + L or MIR + H89, respectively. The slices were then fixed in 4% paraformaldehyde, and thin cryosections (7 μm) were subjected to immunofluorescence and counterstained with Evans blue. ImageJ (version 1.54 b) was used to calculate the fluorescent distribution of the H^+^-ATPase signal along a line starting from the apical membrane and crossing the nucleus along its maximum diameter. The extent of the H^+^-ATPase signal was normalized to the longitudinal length of each cell, which was calculated considering the Evans red signal of the apical and basolateral sides. Tubules with the lumen not clearly visible were excluded from the analysis. For each experimental condition, at least 30 cells were blindly analyzed.

### Cell culture and intracellular pH measurements

Immortalized mouse cortical collecting duct M1 cells (Stoos et al., 1991) were previously stably transfected with human β3-AR and cultured, as previously described (Milano et al., 2018). For intracellular pH measurements, cells were seeded on glass coverslips (Ø 15 mm). Intracellular pH changes were recorded using the ratiometric dye BCECF AM. Cells were incubated with 2.5 μM BCECF for 20 min at 37 °C and then rinsed with saline solution to wash out the extracellularly retained dye. The coverslips with dye-loaded cells were analyzed with a setup that we previously described (Scorza et al., 2023). In brief, during the experiment, Ringer’s solution (containing 140 mM NaCl, 5 mM KCl, 1.2 mM CaCl_2_, 1 mM MgCl_2_, 5 mM glucose, and 10 mM HEPES with a final pH of 7.40) was used to perfuse the cells. BCECF ratios were acquired using a ratio imaging setup running MetaFluor software (version 7.7.3.0, Molecular Devices, San Jose, CA, United States). Each coverslip, mounted in an open-top perfusion chamber, was placed on the heated stage of a Nikon TE200 inverted microscope (Nikon, Tokyo, Japan). For BCECF experiments, cells were alternately excited at 440 nm and 490 nm. The excitation wavelengths were generated by a monochromator system in the path of a 75-W xenon light source. Pairs of fluorescence images for both dyes (emission collected at 520 nm) were captured using a cooled CCD camera CoolSNAP HQ (Photometrics, Tucson, AZ, United States) every 5s and converted to a ratio image using MetaFluor software. We used a specific positive internal control at the end of each run. The BCECF ratio was converted to intracellular pH using a standard calibration procedure based on the use of nigericin in high potassium media buffered at different pH values. For cellular acidification, we used the ammonium-loading removal protocol (Guerra et al., 1993); the NH_4_Cl solution was identical to Ringer’s solution and contained 40 mM NH_4_C1 in addition. A sodium-free (TMA medium) solution was obtained by complete replacement of sodium with tetramethylammonium (TMA). Whenever needed, drugs were added (alone or in combination) to each solution at the following doses: mirabegron (10 nM, β3-AR agonist), L-748 (100 nM, β3-AR antagonist), bafilomycin (10 nM, H^+^-ATPase inhibitor), and H89 (10 μM, PKA inhibitor). The rate of H^+^-ATPase activity was determined upon acidification by calculating the slope of the curve recorded when pH recovery to the basal level occurred. The slope was expressed as ΔpH/min. The slope analysis and the statistical significance of the different experimental conditions were analyzed with one-way ANOVA and Dunnett multiple comparison tests.

### Urine collection and ELISA test for human H^+^-ATPase in urine samples

Quantification of H^+^-ATPase in the 24-h urine samples of patients (N = 12) with an overactive bladder and treated with the human β3-AR agonist mirabegron (Betmiga^®^, Astellas Pharma, Assago, MI, Italy) 50 mg/day was performed using a commercial ELISA kit (catalog # abx385949 Abbexa, https://www.abbexa.com/). H^+^-ATPase was measured in urine samples (100 μL) collected at week -1 (T-1) and 0 (T0) before the initiation of mirabegron treatment and after 4 weeks of treatment. For details about patients and the sample preparation, see Milano et al. (2023). Data of urinary H^+^-ATPase excretion were normalized to the 24-h diuresis (ng/24 h). This study was conducted in accordance with the Declaration of Helsinki and approved by the Ethics Committee of Azienda Ospedaliero-Universitaria “Consorziale Policlinico” (study #4850, protocol code 81130, and date of approval 27 October 2015) for studies involving humans.

### Statistics

All data were analyzed using paired or unpaired Student’s t-test and were previously tested for normality. No data and/or animals were excluded from the analysis. For statistical analysis, GraphPad Prism 9 software (La Jolla, CA, United States) was used. Paired Student’s t-test was used to compare mice of each genotype before and after acid loading. Unpaired Student’s t-test was used to compare CTR and NH_4_Cl groups for either β3-AR^+/+^ or β3-AR^−/−^ mice. The one-way ANOVA multiple comparison test was used for the statistical analysis of *ex vivo* and *in vitro* experiments. All values are expressed as means ± SEM. A difference of *p* < 0.05 was considered statistically significant. Details about statistical analyses are reported in the figure legends.

## Results

### β3-AR is expressed in the kidney collecting duct ICC

**FIGURE 1:**
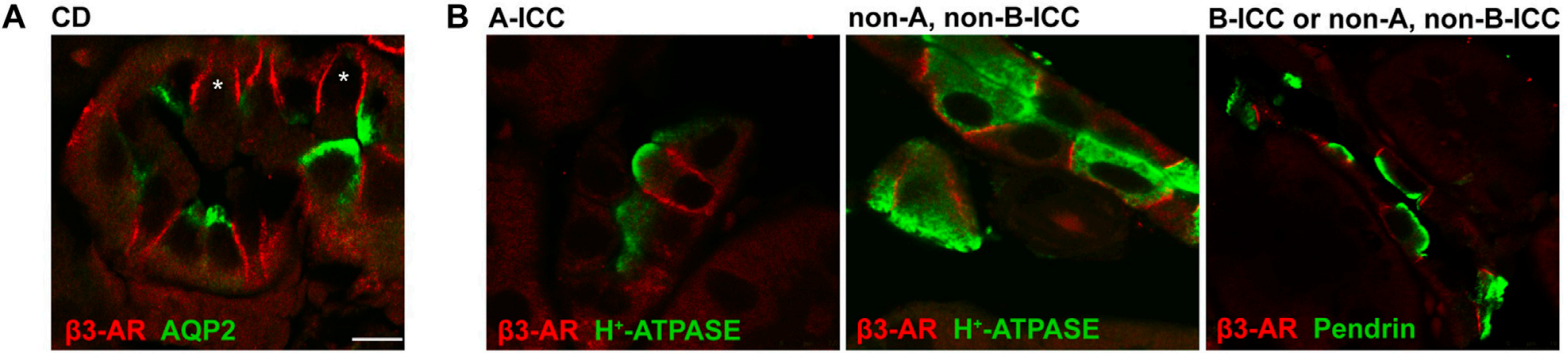
Immunolocalization of β3-AR in mouse kidney intercalated cells. **(A)** Kidney cryosections from wild-type mice were immunostained with anti-β3-AR (red) and anti-AQP2 (green) antibodies. Confocal microscopy showed that the β3-AR receptor was expressed not only in the AQP2-positive principal cells, as we previously reported, but also in the AQP2-negative intercalated cells. **(B)** Kidney cryosections from wild-type mice were immunostained with antibodies against β3-AR (red), H^+^-ATPase, or pendrin (green), which were used as markers of ICC subtypes. Immunofluorescence analysis revealed that β3-AR was expressed at basolateral membrane in A-ICC, identified by the apical expression of H^+^-ATPase, in B-ICC identified by the apical expression of Pendrin, and in nonA, non B cells characterized by apical and diffuse vesicular H^+^-ATPase staining and by pendrin at luminal side. The same results were obtained in five different animals. Scale bar = 8 μm.

The immunolocalization of β3-AR (in red) in mouse collecting ducts revealed that besides the AQP2-positive principal cells, the AQP2-negative cells also expressed β3-AR at the basolateral membrane (Figure 1; see asterisks). To characterize these cells, we used antibodies against either the H^+^-ATPase (in green) or the Cl^−^/HCO_3_^−^ exchanger pendrin (in green). Confocal microscopy revealed that β3-AR was expressed at the basolateral membrane of the A-ICC, identified by the apical expression of H^+^- ATPase (Roy et al., 2015), the B-ICC expressing the apical Pendrin (Roy et al., 2015), and non-A, non- B-ICC characterized by apical and diffuse vesicular H^+^-ATPase staining and Pendrin at the luminal side (Roy et al., 2015). This evidence suggested that β3-AR might be involved in the physiological regulation of ICC activity. Here, we focused on the putative regulatory role of β3-AR in H^+^-ATPase expression and function in mouse kidneys, considering that i) mammals basically cope with acidic dietary intake; ii) the A-ICC is the largest population of ICC (Kim et al., 1999); iii) in the kidney, β3-AR is coupled with Gs protein (Procino et al., 2016), and the cAMP-mediated translocation of the subapical pool of H^+^-ATPases to the apical membrane of A-ICC is a key event occurring during metabolic acidosis (Paunescu et al., 2010).

### Lack of functional β3-AR impairs the H^+^-ATPase expression levels in mouse kidneys

**FIGURE 2:**
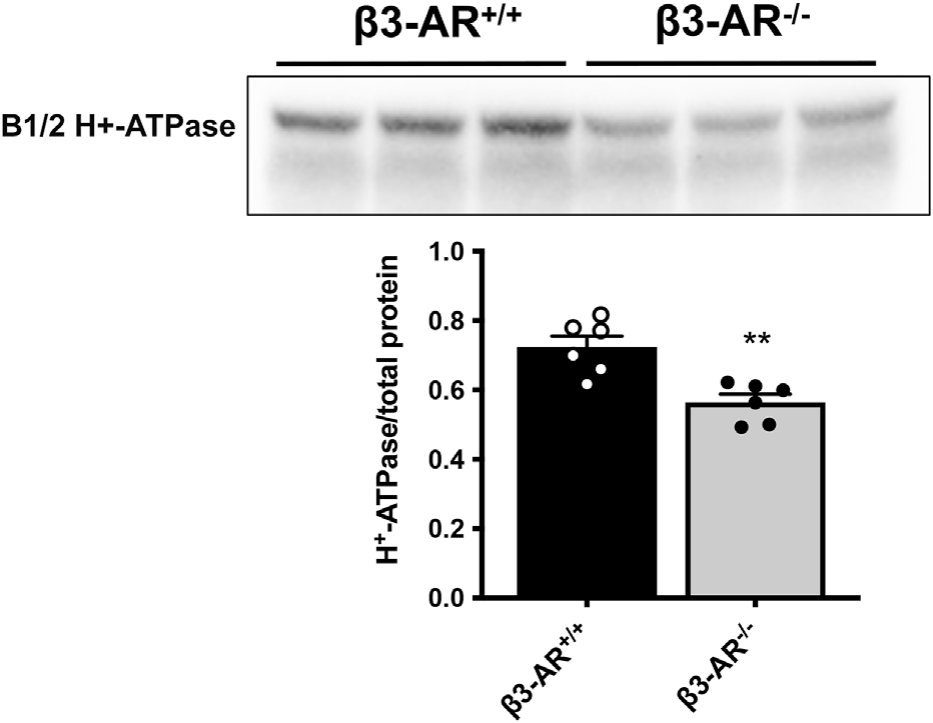
Effect of β3-AR knockout on the H^+^-ATPase expression level in the kidneys. Western blotting with anti-H^+^-ATPase antibodies was carried out using homogenates prepared from whole kidneys of β3-AR^+/+^ (*n* = 6) and β3-AR^−/−^ (*n* = 6) mice. Representative lanes are reported in the figure. The H^+^-ATPase expression levels were normalized to the total protein content using the Stain-free™ gel technology. Densitometric analysis showed a 22% decrease in H^+^-ATPase in β3-AR^−/−^ mice compared to that in β3-AR^+/+^ mice (β3-AR^+/+^: 0.72 ± 0.032 SEM vs. β3-AR^−/−^: 0.56 ± 0.023 SEM). In the plot, each dot corresponds to a mouse, and the bars indicate the SEM. The experiment was repeated three times with comparable results. **p < 0.01 with two-tailed unpaired Student’s *t*-test.

**FIGURE 3:**
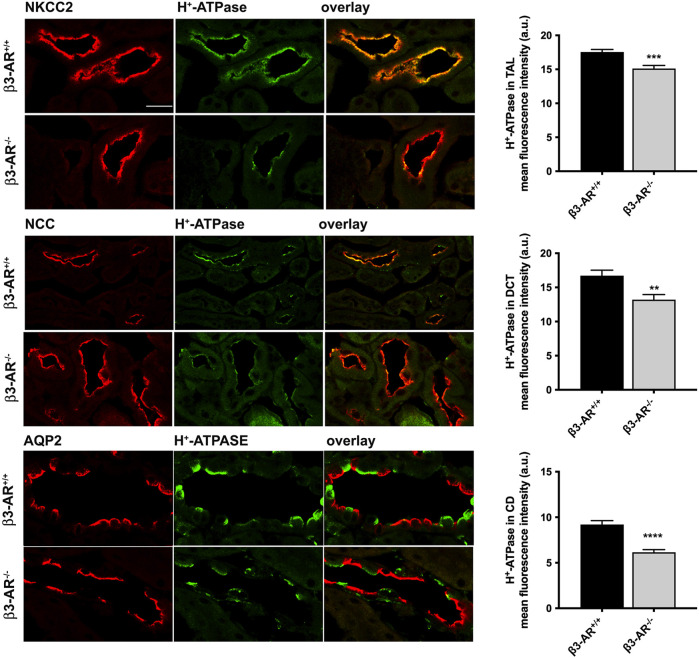
H^+^-ATPase expression level in the thick ascending limb, distal convoluted tubule, and the collecting ducts of β3-AR^−/−^ mice. To evaluate the effect of the β3-AR absence on the H^+^-ATPase expression in the β3-AR-expressing nephron segments, the kidneys from β3-AR^+/+^ and β3-AR^−/−^ mice (*n* = 6 each) were subjected to immunofluorescence experiments using anti-H^+^-ATPase (in green) and anti-NKCC2/NCC/AQP2 (in red) as specific markers of the TAL, DCT, and CD, respectively. Representative images are shown. The experiment was repeated three times, and comparable results were obtained. Scale bar = 25 μm. The analysis of the mean fluorescence intensity (FI) of H^+^-ATPase showed that it was reduced by approximately 20% in the TAL (β3-AR^+/+^FI: 17.55 ± 0.37 SEM vs. β3-AR^−/−^ FI: 15.13 ± 0.44 SEM) and the DCT (β3-AR^+/+^FI: 16.72 ± 0.80 SEM vs. β3-AR^−/−^ FI: 13.2 ± 0.76 SEM) and by approximately 30% in the CD of β3-AR^−/−^ mice compared to that expressed in β3-AR^+/+^ mice (β3-AR^+/+^FI: 9.21 ± 0.42 SEM vs. β3-AR^−/−^ FI: 6.16 ± 0.28 SEM). ***p < 0.001, **p < 0.01, ****p < 0.0001 with two-tailed unpaired Student’s *t*-test.

β3-AR^−/−^ mice were used to unveil a possible role of β3-AR in the H^+^-ATPase regulation. Semi-quantitative Western blotting analysis of total kidney lysates highlighted a 22% reduction in the H^+^-ATPase level in β3-AR^−/−^ mice compared to that in β3-AR^+/+^ mice (Figure 2), suggesting that *in vivo* β3-AR might regulate H^+^-ATPase expression. Since H^+^-ATPase is expressed not only in ICC but also in other renal tubular cells (Brown et al., 1988; Frische et al., 2018), we investigated whether the absence of β3-AR altered the expression of H^+^-ATPase in all cell types where β3-AR is expressed. Quantitative immunofluorescence analysis of H^+^-ATPase expression in β3-AR-positive tubules showed that, in the TAL, identified by NKCC2, H^+^-ATPase expression was significantly reduced to approximately 20% in β3-AR^−/−^ mice compared to that in control mice (Figure 3). The same reduction in H^+^-ATPase expression levels was observed in the DCT, identified by NCC (Figure 3). AQP2 was used as a marker of the final site for the regulation of the acid–base balance, the CD, where H^+^-ATPase expression was reduced to approximately 30% in β3-AR^−/−^ mice compared to that in β3-AR^+/+^ mice (Figure 3). We also investigated renal H^+^-ATPase expression in the double β1,2-AR knockout mice (Rohrer et al., 1999) and their wild-type (WT) controls and found that H^+^-ATPase expression was unchanged, thus suggesting that β1- and β2-AR were not involved in regulating H^+^-ATPase expression (see Supplementary Material S1). These sources of evidence supported the idea that β3-AR positively regulates H^+^-ATPase expression in the kidneys.

### β3-AR knockout mice show a blunted response to acid challenge

**FIGURE 4:**
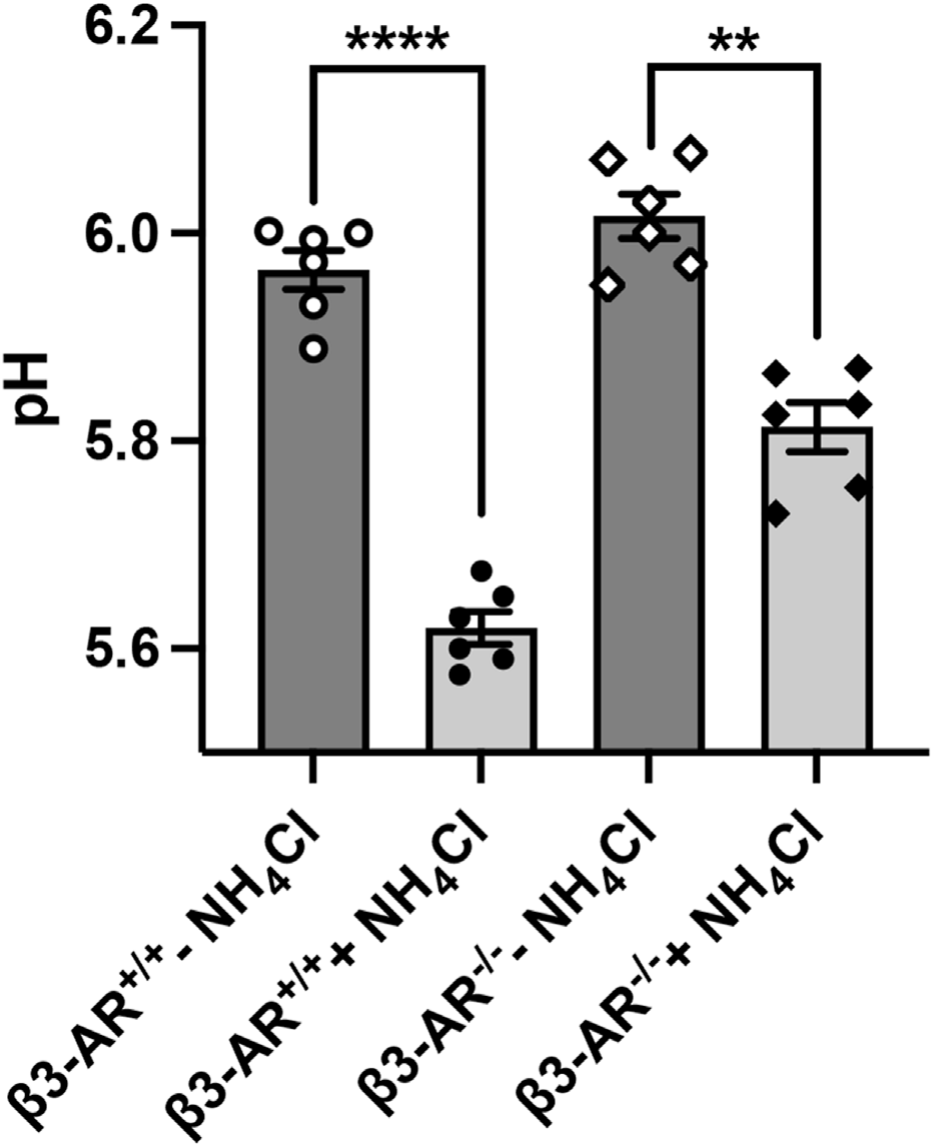
H^+^-ATPase mice exhibit an attenuated response to the acid challenge. β3-AR^−/−^ mice (*n* = 12) and their wild-type β3-AR^+/+^ (*n* = 12) mice were placed in metabolic cages and monitored for 5 days. For each genotype, half of the mice were provided with water (−NH_4_Cl, *n* = 6) and half were provided with a drinking solution containing 280 mM NH_4_Cl for 5 days to induce acid loading (+NH_4_Cl, *n* = 6). The pH of urine, collected over 24 h on the fifth day of treatment, was analyzed and reported in the plot. In both β3-AR^+/+^ and β3-AR^−/−^ mice, the 24-h urine pH decreased after the acid challenge; however, this reduction was blunted in β3-AR^−/−^ mice. For β3-AR^+/+^: ****p < 0.001; for β3-AR^−/−^: ##*p* < 0.01. The graph shows mean ± SEM; each dot represents each animal, and significant differences were calculated with two-tailed paired Student’s t-test. Data were analyzed also by one-way ANOVA performing pairwise comparison between groups: β3-AR^+/+^: ***p < 0.01; for β3-AR^−/−^: ##*p* < 0.01.

To answer the question of whether β3-AR influences the functional activity of H^+^-ATPase, we evaluated the 24-h urine pH of β3-AR^+/+^ and β3-AR^−/−^ mice (Figure 4). Under the basal condition, the urine pH of β3-AR^−/−^ mice was, even though not statistically significant, slightly higher than that of β3-AR^+/+^ mice (β3-AR^−/−^ −NH_4_Cl: 6.016 ± SEM 0.021 vs. β3-AR^+/+^ −NH_4_Cl: 5.965 ± 0.018). This prompted us to examine the response of β3-AR^+/+^ and β3-AR^−/−^ mice to 5 days of acid loading (280 mmol/L NH_4_Cl in drinking water). Strikingly, in β3-AR^−/−^ mice, the drop in urine pH induced by the acid load (Figure 4, +NH_4_Cl) was significantly smaller (ΔpH = 0.203, SEM ±0.034) than that measured in β3-AR^+/+^ mice (ΔpH = 0.35, SEM ±0.027). To evaluate the proton excretion in the urine, we measured titratable acids. Urinary titratable acids were significantly lower in β3-AR^−/−^ mice compared to that in β3-AR^+/+^ mice under basal conditions (β3-AR^−/−^ −NH_4_Cl: 89.33 mmol/L ± SEM 4.57 vs. β3-AR^+/+^ −NH_4_Cl: 109.1 mmol/L ± 4.39, *p* < 0.05). As expected, both β3-AR^+/+^ and β3-AR^−/−^ mice showed an increased excretion of urine titratable acids after acid loading. However, this increase was significantly blunted in β3-AR^−/−^ mice compared to β3-AR^+/+^ mice (β3-AR^−/−^ + NH_4_Cl: 107 mmol/L ± SEM 1.86 vs. β3-AR^+/+^ +NH_4_Cl: 141 mmol/L ± 4.49, *p* < 0.001), suggesting that β3-AR is required for proton elimination in the urine. However, no statistically significant differences between β3-AR^+/+^ and β3-AR^−/−^ mice were observed in NH_4_^+^ excretion in urine, neither under basal conditions (β3-AR^−/−^ −NH_4_Cl: 50.67 mmol/L ± SEM 4.65 vs. β3-AR^+/+^ −NH_4_Cl: 56.27 mmol/L ± 7.77) nor after acid loading (β3-AR^−/−^ +NH_4_Cl: 188.5 mmol/L ± SEM 3.99 vs. β3-AR^+/+^ +NH_4_Cl: 182.7 mmol/L ± 4.37).

In addition, to exclude the contribution of the environmental factors or stress related to the metabolic cage housing, we also compared, for each genotype, the pH of urine collected in parallel from mice that were provided with water containing NH_4_Cl and mice given only water and found comparable results (Table 1). We measured also the water intake and we found that, as expected, it was reduced in NH4Cl-watered mice compared to control mice for both genotypes (see Table 1). Consistent with our previous observation in β3-AR^−/−^ mice, the water intake was significantly higher compared to that in β3-AR^+/+^ mice, likely to compensate for their mild polyuria (Procino et al., 2016), and it was also evident after acid loading.

**TABLE 1:** Metabolic data of β3-AR^+/+^ and β3-AR^−/−^ mice before and after 5 days of acid loading. Paired Student’s t-test was used to compare mice of each genotype before (−NH_4_Cl) and after acid loading (+NH_4_Cl). Unpaired Student’s t-test was used to compare CTR and NH_4_Cl groups for either β3-AR^+/+^ or β3-AR^−/−^ mice. All values are expressed as means ± SEM. β3-AR^+/+^: A) monitoring vs. after 5 days: **p < 0.05, ***p < 0.001, ****p < 0.0001; B) CTR vs. NH_4_Cl: $ *p* < 0.05, $$ *p* < 0.01, $$$$ *p* < 0.0001. β3-AR^−/−^: A) monitoring vs. after 5 days: #*p* < 0.05, ##*p* < 0.01; B) CTR vs. NH_4_Cl: °°*p* < 0.01, °°°*p* < 0.001. The urine output of β3-AR^−/−^ mice was always significantly higher than that of β3-AR^+/+^ mice.

			Urine pH	Urine output (mL)	Plasma pH	Water intake (mL)	Food intake (g)	Body weight (g)
β3-AR^+/+^	CTR (n = 6)	Monitoring	5.97 ± 0.018	1.20 ± 0.032	7.36 ± 0.02	5.45 ± 0.374	5.04 ± 0.123	28.74 ± 0.530
		After 5 days	5.99 ± 0.017	1.00 ± 0.120	7.29 ± 0.03	5.45 ± 0.305	4.63 ± 0.16	28.35 ± 0.653
	NH_4_Cl (n = 6)	Monitoring (−NH_4_Cl)	5.965 ± 0.019	1.30 ± 0.110	7.32 ± 0.010	5.21 ± 0.252	4.78 ± 0.175	28.65 ± 0.547
		After 5 days (+NH_4_Cl)	5.62 ± 0.016^****;^^$$$$^	1.25 ± 0.180	7.19 ± 0.020^***;$^	3.605 ± 0.341^**;^^$$^	4.393 ± 0.197	28.25 ± 0.653
β3-AR^−/−^	CTR (n = 6)	Monitoring	6.01 ± 0.019	2.00 ± 0.150	7.34 ± 0.01	6.7 ± 0.234	4.59 ± 0.226	29.20 ± 0.563
		After 5 days	6.02 ± 0.024	1.80 ± 0.200	7.35 ± 0.03	6.65 ± 0.25	4.81 ± 0.22	28.70 ± 0.522
	NH_4_Cl (n = 6)	Monitoring (−NH_4_Cl)	6.016 ± 0.021	1.97 ± 0.050	7.31 ± 0.02	6.60 ± 0.277	4.59 ± 0.226	28.67 ± 0.587
		After 5 days (+NH_4_Cl)	5.813 ± 0.024^##,^^°°°^	1.90 ± 0.078	7.20 ± 0.02^##,^^°°^	5.48 ± 0.249^#,^^°^	4.20 ± 0.224	29.10 ± 0.587

### In kidney slices, the β3-AR stimulation promotes H^+^-ATPase apical expression

**FIGURE 5:**
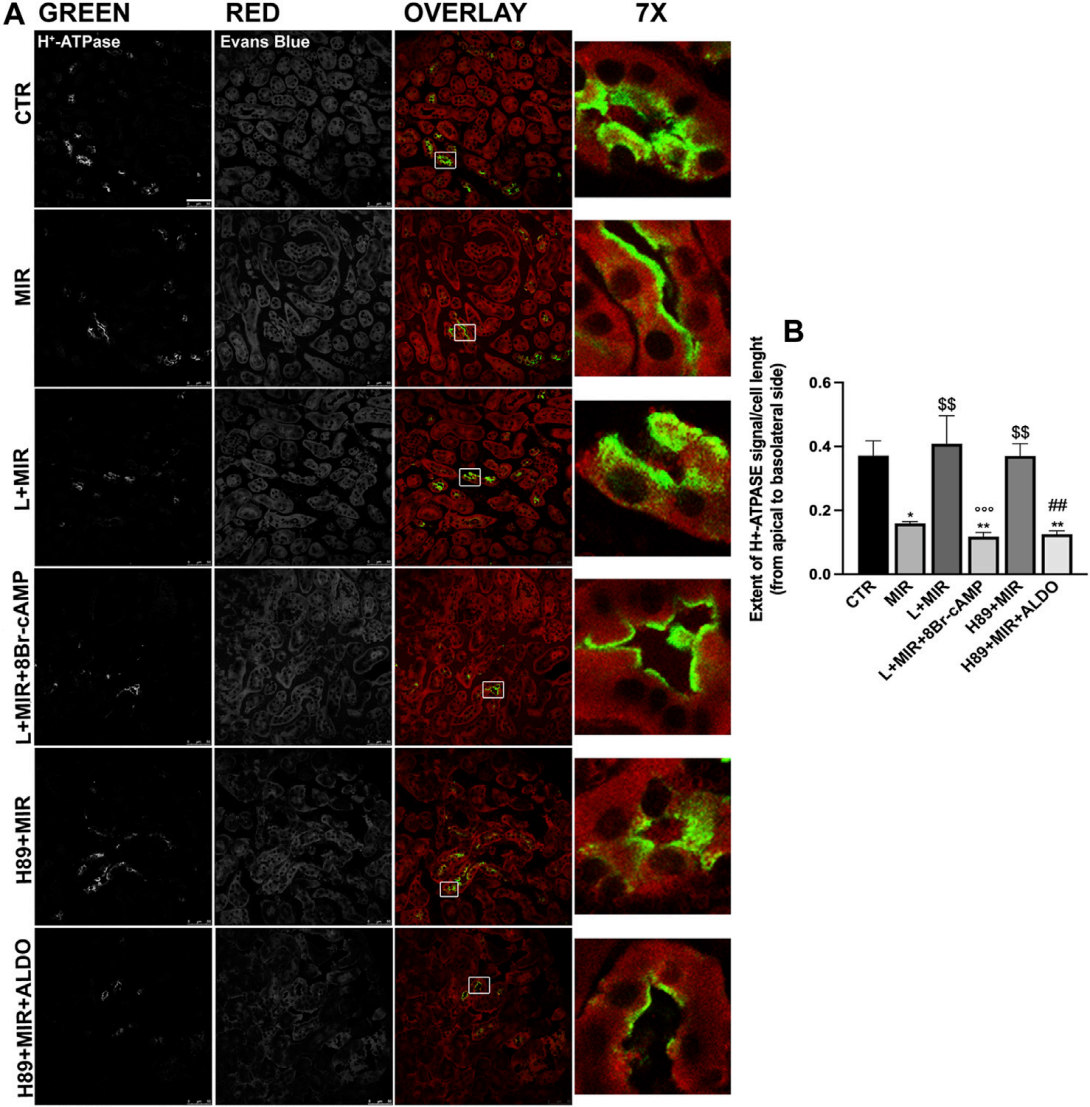
*Ex vivo* β3-AR stimulation promotes H^+^-ATPase apical expression in the kidney slices. **(A)** Freshly isolated kidney slices (250 μm thick) from six WT mice were left untreated (CTR) or incubated for 40 min in a complete culture medium with the β3-AR agonist mirabegron (10^–8^ M) alone or after 30 min of preincubation with either the β3-AR antagonist (L748,337, 10^–7^ M) or the PKA inhibitor (H89, 10^–5^ M). As positive controls of the cell responsiveness, 8-bromo-cAMP (5 × 10^−4^ M) or aldosterone (ALDO, 2 × 10^−7^ M) were used to activate H^+^-ATPase regardless of the β3-AR/PKA pathway. Slices were then fixed, and ultrathin cryosections (7 μm) were stained with antibodies against H^+^-ATPase (green), counterstained with Evans blue (red), and analyzed by confocal microscopy. MIR promoted H^+^-ATPase accumulation at the apical plasma membrane of renal tubular cells compared with the punctate intracellular staining of H^+^-ATPase observed in untreated samples (CTR) or slices stimulated with MIR after preincubation with L748,337 or H89. Images are representative of three independent experiments. Scale bar = 50 μm. **(B)** Histogram summarizes the effects of the treatments on the extent of the H^+^-ATPase signal from the apical to the basolateral side. For each experimental condition, at least 30 cells were blindly analyzed. The graph shows mean ± SEM, and significant differences were calculated with respect to the control condition by ordinary one-way ANOVA and Tukey’s multiple comparison test. *: vs. CTR, *p < 0.05; **p < 0.01; $ vs. MIR: $$*p* < 0.01; °L + MIR vs. L + MIR + 8Br-cAMP: °°°*p* < 0.001; #H89 + MIR vs. H89 + MIR + ALDO: ##*p* < 0.01.

The finding that the absence of functional β3-AR reflected in the reduction of H^+^-ATPase expression and function *in vivo* prompted us to more deeply investigate the effect of the β3-AR activation on both the H^+^-ATPase subcellular localization in live kidney slices and the H^+^-ATPase activity in a mouse kidney cell line. Confocal microscopy of the kidney slices from wild-type mice showed that the β3-AR agonist mirabegron promoted the accumulation of H^+^-ATPase at the apical plasma membrane of tubular epithelial cells compared with the cytoplasmic localization of H^+^-ATPase observed in untreated slices (Figure 5A). This effect of mirabegron was prevented by preincubation either with the β3-AR antagonist L748,337 or with the PKA inhibitor H89, confirming that the effect of mirabegron was ascribable to β3-AR stimulation and involved PKA. As positive controls of the cell responsiveness, 8-bromo-cAMP was used in the presence of L + MIR, and aldosterone was added to H89 + MIR to activate the H^+^-ATPase through the PKC pathway (Roy et al., 2013). The mirabegron effect was attributable to β3-AR stimulation since mirabegron failed to induce H^+^-ATPase apical expression in the kidney of β3-AR^−/−^ mice (Supplementary Material S2). As shown in Figure 5B, the analysis of the extent of the H^+^-ATPase fluorescent signal within the cell from the apical to the basolateral side indicated that upon β3-AR activation, H^+^-ATPase was preferentially localized at the apical plasma membrane rather than in the cytosol. Pretreatments with the β3-AR antagonist or PKA inhibitor maintained the extent of the H^+^-ATPase fluorescent signal in the cytosol as under the control condition. Treatment with 8Br-cAMP or aldosterone in the presence of L748,337 or H89, respectively, promoted H^+^-ATPase apical accumulation, as expected.

### β3-AR stimulation promotes the H^+^-ATPase activity *in vitro* in renal cells

**FIGURE 6:**
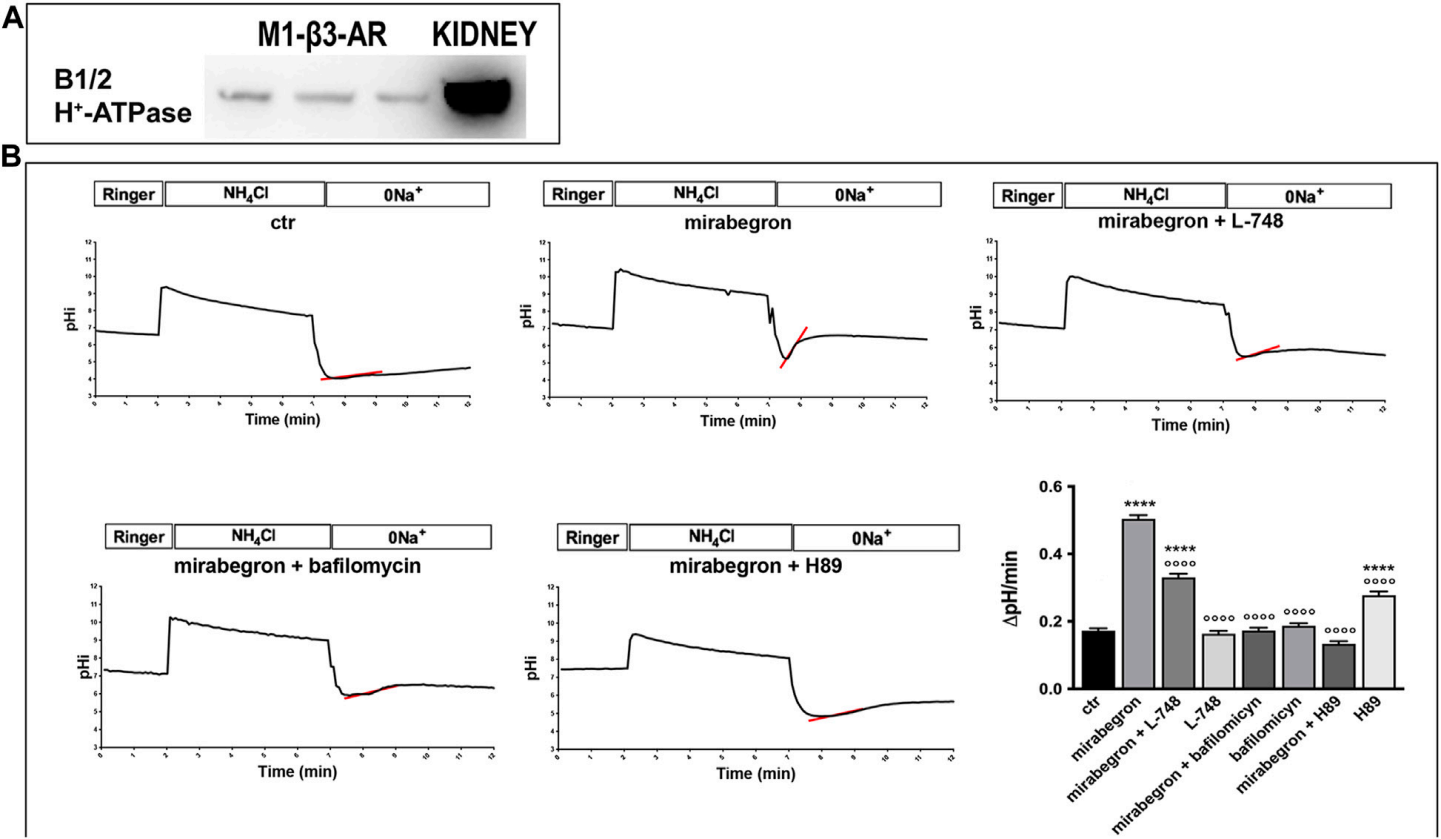
*In vitro* β3-AR activation promotes H^+^-ATPase activity in mouse renal epithelial cells. **(A)** M1-β3-AR cells, stably transfected with human β3-AR, express endogenous H^+^-ATPase, as indicated by the Western blotting experiments. Total kidney lysate was used as the positive control. **(B)** M1-β3-AR cells, loaded with the ratiometric pH-sensitive dye BCECF, were acidified using ammonium chloride prepulse. The rate of H^+^-ATPase activity was determined as the alkalinization rate of the intracellular pH in the absence of sodium. Treatment with mirabegron, a human β3-AR agonist, increased the intracellular pH recovery rate by approximately 2.5-fold compared to that of untreated cells. This effect was either prevented by the β3-AR antagonist L-748 or by the PKA inhibitor H89, which block the cAMP-mediated pathway coupled with β3-AR. The pH recovery rate was attributable to H^+^-ATPase since it was abolished by its inhibitor bafilomycin. ****p < 0.001 vs. CTR, °°°°*p* < 0.001 vs. mirabegron. The graph shows mean values ± SEM, and significant differences were calculated by ordinary one-way ANOVA and Dunnett’s multiple comparison test.

To study in detail the possible regulatory effect of β3-AR on the activity of H^+^-ATPase, we exploited a mouse renal cell line M1-CCD showing a mixed phenotype between principal and ICCs, expressing endogenous H^+^-ATPase, and stably transfected with human β3-AR (M1-β3-AR; Figure 6A). The cells, which were loaded with the ratiometric pH-sensitive dye BCECF, were acidified by the NH_4_Cl prepulse technique and allowed to recover their intracellular pH over time by extruding protons through endogenously expressed proton transporters and pumps. To exclude the contribution of the sodium-proton exchanger (NHE) to this compensatory response and directly correlate it with the H^+^-ATPase activity, cells were washed out and maintained in a Na-free bathing solution. The activity of H^+^-ATPase was measured as the rate of intracellular pH (pHi) recovery in the absence of extracellular Na^+^ after an NH_4_Cl prepulse. When compared to unstimulated cells, treatment with the β3-AR agonist mirabegron increased the intracellular pH recovery rate by approximately 2.5-fold, demonstrating that β3-AR activation stimulates H^+^-ATPase activity (Figure 6B). This effect was strictly related to the β3-AR activation since it was prevented by either the β3-AR antagonist L-748,337 or the PKA inhibitor H89. In addition, the pH recovery rate was abolished by the H^+^-ATPase inhibitor bafilomycin. These results demonstrate that β3-AR stimulation promotes proton extrusion by activating H^+^-ATPase.

### Human ICCs express β3-AR and its stimulation increases urinary excretion of H^+^-ATPase

**FIGURE 7:**
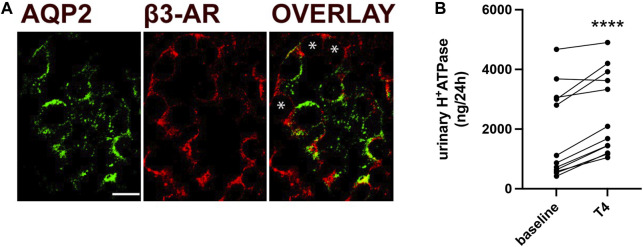
In humans, β3-AR stimulation increases urinary H^+^-ATPase excretion. **(A)** Human kidney sections were stained with antibodies against β3-AR (red) and AQP2 (green), which were used as markers of CD principal cells. The overlay shows the expression of β3-AR at the basolateral membrane of AQP2-positive principal cells and AQP2-negative ICCs, indicated by asterisks. Scale bar = 10 μm. **(B)** ELISA measurements of H^+^-ATPase excreted in 24-h urine samples collected from patients treated with the β3-AR agonist mirabegron for the treatment of overactive bladder syndrome. Analysis was performed at baseline (T1 and T0 before the treatment) and after 4 weeks of treatment. Data were normalized to the 24-h urine volume. Urinary H^+^-ATPase excreted showed an increase of approximately 35%. N = 12 patients. The values obtained were compared using the paired t-test. Data are reported as mean ± SEM. ****p < 0.0001.

To further determine the relevance of our findings in human context, we examined β3-AR localization in human ICCs. Here, we showed that in human CD, β3-AR is expressed at the basolateral membrane of AQP2-negative ICCs, indicated by asterisks in Figure 7A, as well as in AQP2-positive principal cells, as shown already (Milano et al., 2023). To investigate whether β3-AR stimulation might positively affect the trafficking of H^+^-ATPase in human tubular cells, we measured urinary H^+^-ATPase excretion in patients treated with mirabegron (Betmiga^®^) for an overactive bladder syndrome. The excretion of H^+^-ATPase, quantified in 24-h urine samples using an ELISA test, was normalized to the 24-h urine volume. Analysis was performed before initiation of mirabegron treatment (baseline) and after 4 weeks of treatment. Of note, mirabegron treatment increased the amount of the proton pump excreted in the urine samples by approximately 35%, indicating that β3-AR regulates H^+^-ATPase trafficking in humans also (Figure 7B).

## Discussion

The purpose of this study was to understand the involvement of β3-AR in regulating the activity of H^+^-ATPase in the kidneys. The kidney plays a pivotal role in acid–base homeostasis; however, the role of the sympathetic nervous system in controlling acid–base balance has been poorly studied. Even though it has been reported that the β-AR-agonist isoproterenol is involved in the regulation of ICC functions (Morel et al., 1976; Iino et al., 1981; Morel and Doucet, 1986; Hayashi et al., 1991; DiBona and Kopp, 1997), the role of the sympathetic nervous system in controlling acid–base balance still needs to be elucidated. In addition, so far, the role of the sympathetic control of renal functions has been described taking into account only the expression of β1- and β2-AR since the expression of β3-AR in the kidneys has only recently been discovered (Procino et al., 2016). In particular, we demonstrated that in mouse kidneys, β3-AR is expressed in most of the nephron segments also expressing the type-2 vasopressin receptor and that its stimulation increases tubular reabsorption of water and solutes by acting on NKCC2, NCC, and AQP2 (Procino et al., 2016; Milano et al., 2021). An LC-MS/MS-based proteomic analysis carried out in H^+^-ATPase-rich cells revealed β3-AR expression in ICCs (Da Silva et al., 2010). However, no information has been reported on the subcellular localization and function of β3-AR in ICCs.

Herein, we report, for the first time, the localization of β3-AR in all types of renal ICC, which are responsible for the final tuning of urine acidification in the CD (Figure 1). Considering that mammals basically cope with acidic dietary intake and that the mechanism of acidosis compensation by A-ICC involves active proton secretion mainly via the H^+^-ATPase, and less importantly via the K^+^-H^+^-ATPase, in this study, we focused on unveiling a possible link between β3-AR activation and H^+^-ATPase expression/activity. To evaluate the effect of the β3-AR absence on the H^+^-ATPase expression and function, we exploited β3-AR^−/−^ mice. Western blotting analysis showed that the H^+^-ATPase expression was significantly reduced in the kidneys of β3-AR^−/−^ mice compared to those of β3-AR^+/+^ mice (Figure 2). On the contrary, the renal H^+^-ATPase abundance was unchanged in β1,2-AR knockout mice compared to their wild-type counterpart (Supplementary Material S1). Boivin et al. (2001) reported β1-AR apical expression in the TAL, all portions of distal tubule nephron segments, A-ICC, and juxtaglomerular and mesangial cells and proximal tubules and β2-AR apical localization in the proximal tubules and, to a lesser extent, in the DCT and CD. Considering the β1- and β2-AR expression in the tubular segments involved in the acid–base homeostasis, it might be important to elucidate *in vivo* their role in controlling acid–base balance. Moreover, the fact that the H^+^-ATPase expression was not modified in β1,2-AR knockout mice does not exclude the possible role of β1- and β2-AR in modulating H^+^-ATPase activity and needs further investigation. Of note, in β3-AR^−/−^ mice, the H^+^-ATPase expression was reduced not only in the CD ICC but also in the TAL and the DCT tubular cells (Figure 3). In this regard, patients with loss-of-function mutations in the gene encoding for the B1 subunit of the H^+^-ATPase, present with a severe clinical phenotype, cannot be explained by dysfunction of A-ICC alone (Gueutin et al., 2013), thus suggesting an important contribution of H^+^-ATPase in other ICC types and/or epithelial cells of the TAL and the DCT (Frische et al., 2018). Therefore, our results, by unveiling the role of β3-AR in regulating the H^+^-ATPase, are important for the in-depth understanding of the complex regulation of proton secretion under physiological and pathological conditions.

Importantly, β3-AR^−/−^ mice showed a reduced ability to excrete protons when challenged by an acid load compared to β3-AR^+/+^ mice, suggesting that β3-AR activity is required to facilitate proton excretion (Figure 4). This was demonstrated by the analysis of urine titratable acids, which were significantly lower in β3-AR^−/−^ compared with those in β3-AR^+/+^ mice under basal conditions and even more evident after acid loading. On the other hand, the urine excretion of NH_4_^+^, an important system for luminal proton buffering, was the same between β3-AR^−/−^ and β3-AR^+/+^ mice under basal conditions and increased with no differences between the two genotypes after acid loading.

Even though the proximal tubule is the main site of ammoniagenesis and luminal secretion of both NH_3_ and NH_4_^+^ (Weiner and Verlander, 2011), this nephron segment does not express β3-AR (Procino et al., 2016); thus, the NH_4_^+^ production of the proximal tubule should not differ between the two genotypes.

Instead, in the TAL, DCT, and CD of β3-AR^−/−^mice, the reduced expression of the H^+^-ATPase should result in decreased proton luminal secretion, leading to a reduction in both titratable acids and protonation of the CD-secreted NH_3_. It is to be considered that NKCC2 can reabsorb a significant amount of NH_4_^+^ in place of K^+^ from the filtrate (Good, 1994). However, in the β3-AR^−/−^ mice, the reduced NKCC2 activity (Procino et al., 2016) would increase the delivery of NH_4_^+^ to the CD, partially counteracting the reduced NH_4_^+^ formation in the CD. Collectively, the sum of i) an increased NH_4_^+^ delivery from the TAL and ii) a reduced NH_4_^+^ formation in the CD might explain the lack of an expected reduction in the urine NH_4_^+^ excretion in the β3-AR^−/−^ mice.

To confirm the β3-AR involvement in the H^+^-ATPase regulation, we stimulated *ex vivo* vital mouse kidney slices with a β3-AR agonist. We found that β3-AR activation with mirabegron promoted H^+^-ATPase apical accumulation in β3-AR-expressing epithelial cells along the nephron, and this was prevented by the β3-AR antagonist L748,337 or the PKA inhibitor H89 (Figure 5). Mirabegron failed to induce H^+^-ATPase apical expression in the kidneys of β3-AR^−/−^ mice, demonstrating the role of β3-AR in regulating the H^+^-ATPase localization (Supplementary Material S2). To study in detail the effect of β3-AR stimulation on the H^+^-ATPase activity, we employed a mouse kidney cell line stably expressing β3-AR. In this cell model, the β3-AR agonism clearly increased the H^+^-ATPase activity and that effect was prevented by β3-AR antagonism or PKA inhibition (Figure 6). The stimulatory role of cAMP in H^+^-ATPase apical recruitment and activation in A-ICC has already been reported (Gong et al., 2010; Paunescu et al., 2010). Previous works reported that, in A-ICC, apical H^+^-ATPase accumulation is elicited by luminal bicarbonate via a soluble adenylate cyclase (sAC)-mediated increase in intracellular cAMP (Paunescu et al., 2010). cAMP could also be generated inside these cells through other signaling pathways. Indeed, ANG II also stimulates proton secretion by A-ICC and induces apical H^+^-ATPase accumulation in the cortical CD (Pech et al., 2008). Aldosterone also induces apical H^+^-ATPase accumulation in ICC of the outer medullary CD in a PKC-dependent manner (Winter et al., 2004). Here, we provide the evidence that the cAMP–PKA pathway, activated by β3-AR stimulation, is involved in renal acid–base homeostasis by regulating the expression, trafficking, and activity of H^+^-ATPase in all the β3-AR-expressing nephron segments. However, we did not investigate the molecular mechanism responsible for renal H^+^-ATPase downregulation in the absence of functional β3-AR. The cAMP pathway-elicited β3-AR stimulation might activate CREB signaling and transcription. Indeed, Kluge et al. (2019) described CREB binding sequences in the promoter regions of the human B1 subunit of the V1 domain of H^+^-ATPase (ATP6V1B1) in the human submandibular cell line. This aspect deserves further investigation.

The study of the trafficking and function of the solute channels and transporters in humans is limited because of the impossibility of carrying out functional experiments. For several apical membrane proteins, including NKCC2 and AQP2, it has been described that their release into the urine is increased by hormonal or pharmacologic stimulations increasing their apical plasma membrane expression (Pisitkun et al., 2004; van der Lubbe et al., 2012; Higashijima et al., 2013; Milano et al., 2023). Of note, several subunits of H^+^-ATPase were detected by LC-MS/MS in human urinary exosomes (Gonzales et al., 2009), and B1 and B2 subunits have been specifically detected in human urinary exosomes by immunoblotting (Pathare et al., 2018). Therefore, we investigated whether the stimulation of β3-AR affected the H^+^-ATPase urinary excretion in humans.

We found that in overactive bladder patients treated with mirabegron, the β3-AR stimulation increased the urinary excretion of H^+^-ATPase (Figure 7B). Even though the experimental proof for the hypothesis that urine H^+^-ATPase was directly proportional to H^+^-ATPase at the apical membrane of epithelial cells in humans is missing, we speculate that during the β3-AR stimulation, an increase in H^+^-ATPase in the plasma membrane that we observed in mice also occurs in the human kidneys.

The urine H^+^-ATPase excretion could also derive from the luminal membrane of the bladder epithelium, where H^+^-ATPase (Tomochika et al., 1997) and β3-AR (Otsuka et al., 2008) are expressed.

However, it is reasonable to assume that the majority of the H^+^-ATPases excreted in the urine is of tubular origin given that the total surface of the tubular epithelium along all segments of the nephrons (on average of 2 million in an adult (Nyengaard and Bendtsen, 1992; Hoy et al., 2003)) that express both H^+^-ATPase and β3-AR is probably of an higher order of magnitude compared with the surface of the bladder epithelium.

Collectively, these results support our hypothesis that β3-AR, as a target of the sympathetic nervous system, is crucial in regulating H^+^-ATPase activity. We envision that H^+^-ATPase modulation by the sympathetic stimulation of β3-AR may play a physiological role in regulating acid–base homeostasis. We further envision that the activation of β3-AR via the sympathetic nervous system could regulate the tubular functions in an integrated way. In particular, during increased physical activity, sympathetic activation of β3-AR triggers an antidiuretic response, as previously described (Procino et al., 2016), where increasing water and electrolyte tubular reabsorption would, in turn, increase the blood pressure, thus supporting muscle work. In parallel, as we hypothesized here, the activation of β3-AR in the TAL, DCT, and CD might also facilitate the urine excretion of proton excess generated during muscle work in the form of lactic acid and carbonic acid. Taken together, these results shed new light on a novel physiological role of β3-AR in regulating acid–base homeostasis and have important possible clinical implications for correcting altered metabolic states, such as acidosis.

## Data Availability

The raw data supporting the conclusion of this article will be made available by the authors, without undue reservation.
