# Supplementary material for: β3-Adrenoceptor as a new player in the sympathetic regulation of the renal acid–base homeostasis
Source: Front Physiol. 2024 Feb 22;15:1304375. doi: 10.3389/fphys.2024.1304375 (PMC10917900; doi:10.3389/fphys.2024.1304375)
Supplement: Supplementary file 1 [file DataSheet1.docx]

Supplementary Material

**
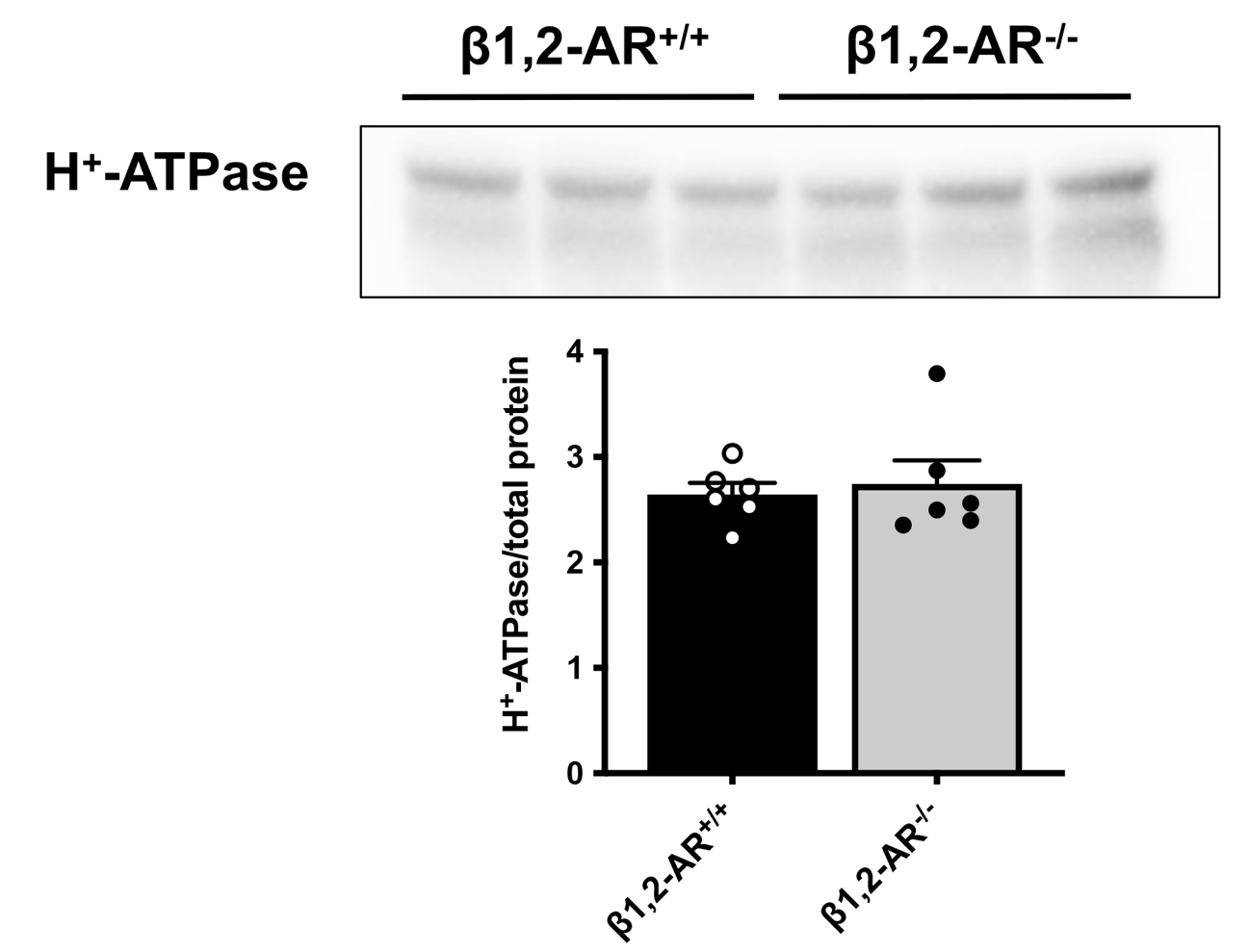
**

**Supplemental S1. Renal H^+^-ATPase expression level is not altered by the absence of β1,2-AR receptors**

Western blotting with anti- B1/2 H^+^-ATPase antibodies was carried out using homogenates prepared from whole kidneys of β1,2-AR^+/+^ (n=6) and β1,2-AR^-/-^ (n=6) mice (Rohrer et al., 1999). Representative lanes were reported in the figure. The H^+^-ATPase expression levels were normalized to total protein content using Stain-free™ gels technologies. Densitometric analysis showed comparable H^+^-ATPase expression levels in both genotypes. In the plot, each dot corresponds to each mouse and the bars indicate the SEM. The experiment was repeated three times and comparable results were obtained. Statistical analysis was performed by two-tailed unpaired Student’s t-test.

ROHRER, D. K., CHRUSCINSKI, A., SCHAUBLE, E. H., BERNSTEIN, D. & KOBILKA, B. K. 1999. Cardiovascular and metabolic alterations in mice lacking both beta1- and beta2-adrenergic receptors. *J Biol Chem,* 274**,** 16701-8.

**
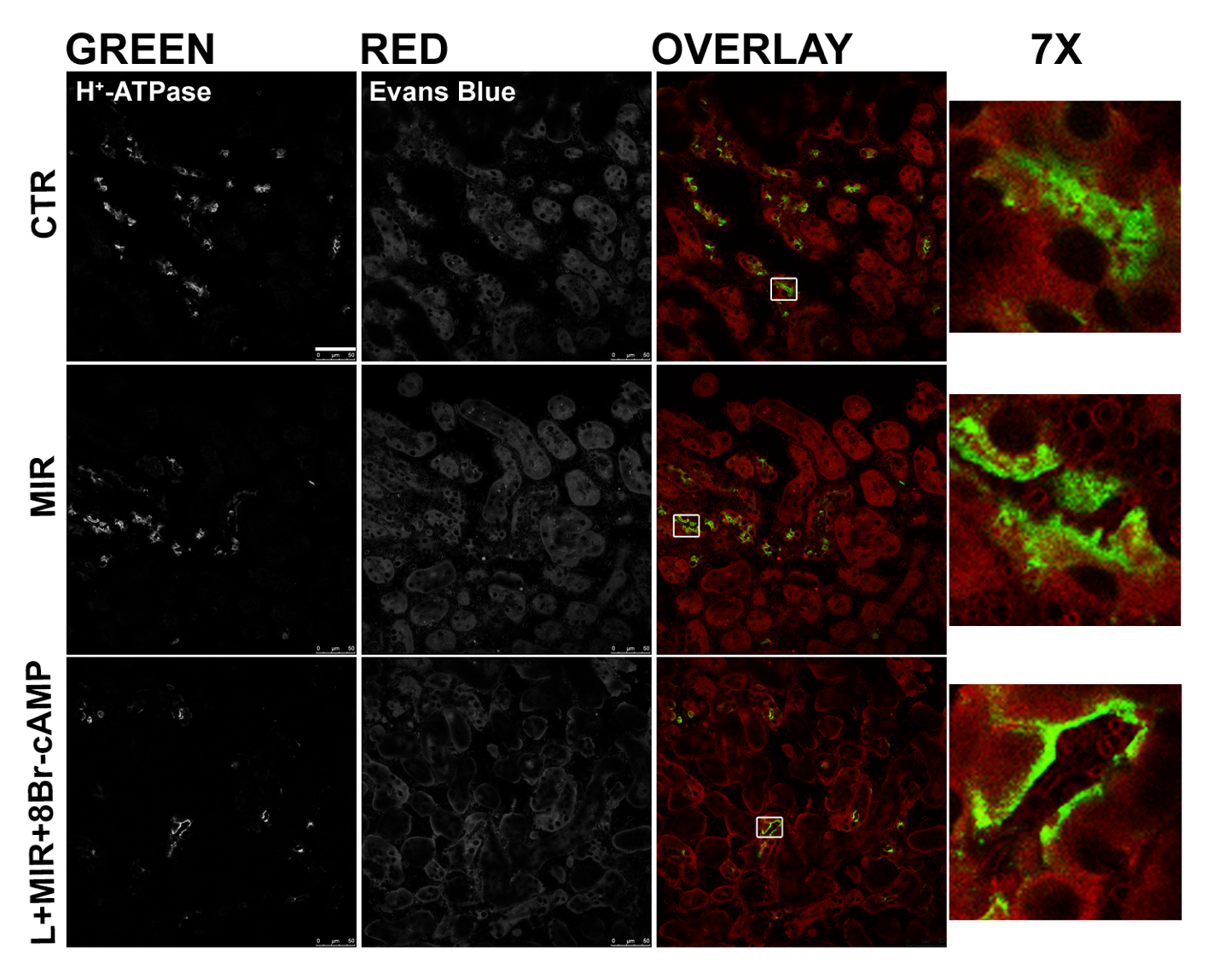
**

**Supplemental S2. β3 stimulation failes to increase H^+^-ATPase apical expression in kidney of β3-AR^-/-^ .** Freshly isolated kidney slices (250 μm thick) from 3 β3-AR^-/-^ mice were left untreated (CTR) or incubated for 40 min in complete culture medium with the β3-AR agonist Mirabegron (MIR, 10^-8^ M). As positive controls of the cell responsiveness, 8-Bromo-cAMP (8Br-cAMP, 5x 10^-4^ M) was used to promote H^+^-ATPase apical expression. Slices were fixed and ultrathin cryosections (7 μm) were stained with antibodies against H^+^-ATPase (green), counterstained with Evans blue (red) and subjected to confocal laser-scanning microscopy. In β3-AR^-/-^ mice, MIR was unable to promote H^+^-ATPase apical expression. Scale bar= 50 μm.
